# Catamenial Pneumothorax in a Patient with Endometriosis: A Case Report

**DOI:** 10.7759/cureus.42193

**Published:** 2023-07-20

**Authors:** Mary Rometti, Laryssa Patti

**Affiliations:** 1 Emergency Medicine, Rutgers Robert Wood Johnson Medical School, New Brunswick, USA

**Keywords:** pelvic hematoma, hemothorax, catamenial pneumothroax, endometriosis, abdominal pain, thoracic endometriosis

## Abstract

Pelvic pain is a common presentation to the emergency department (ED). For female patients, endometriosis can be difficult to diagnose and can have life-threatening complications if missed. In this case report, we present a case of a patient initially presenting to the ED with a few days of crampy lower abdominal pain. After initial imaging, she was found to have a large pelvic hematoma with concern for active extravasation and a large hemothorax. After further evaluation, she was suspected of having endometriosis leading to thoracic endometriosis and a catamenial pneumothorax. Although endometriosis is not typically an emergent diagnosis, the complications of significant endometrial tissue spread can cause life-threatening impacts. Clinicians should consider complications of endometriosis in females of menstruating age.

## Introduction

Pelvic pain is a common reason for female patients to present to the emergency department (ED) [1]. Although it is difficult to know the exact prevalence rates of endometriosis, about 176 million females globally are estimated to have endometriosis [2]. Endometriosis is the presence of endometrial tissue in the body beyond the uterus [2-4]. Patients may present with chronic pelvic pain, dysmenorrhea, urinary frequency or urgency, dysuria, constipation, dyspareunia, or infertility [3,5]. Given its nonspecific presentation and similarity to other diagnoses, endometriosis is frequently difficult to diagnose, with definitive diagnosis confirmed during surgery [2,5]. At times, the delay in diagnosis of endometriosis can be up to 6.7 years [2,5].

The most common presentation of endometriosis is pelvic endometriosis, followed by thoracic endometriosis [3,6]. Thoracic endometriosis occurs when endometrial tissue is located in the lungs or pleura [4]. Clinically, the most common presenting form of thoracic endometriosis is a catamenial pneumothorax [3], defined as reoccurring air in the pleural cavity in reproductive-age females without another pulmonary diagnosis [7].

In this case report, we present an instance in which a patient with a suspected past medical history of catamenial pneumothorax presents with a massive atraumatic hemothorax.

## Case presentation

A 38-year-old female presented to the ED for evaluation of three to four days of lower abdominal cramps. She had previously been seen by her primary doctor and diagnosed with gastroenteritis, attributed to eating at a seafood restaurant prior to the onset of symptoms. She was prescribed ondansetron, which she had been taking without significant relief. She denied chest pain, shortness of breath, and fever. The first day of her last menstrual period was approximately seven days prior to her arrival in the ED.

Her past medical history was significant for a remote history of ovarian cyst as a teenager. She is nulliparous. At age 35, she had a spontaneous right pneumothorax that persisted after chest tube placement and ultimately required video-assisted thoracic surgery (VATS), wedge resection, pleurodesis, and diaphragm plication. The patient was unable to provide significant information regarding this procedure and its etiology.

During this ED visit, her initial vitals were temperature of 36.6°F, heart rate of 68 beats per minute, blood pressure of 106/58 millimeters of mercury (mmHg) , respiratory rate of 16 breaths per minute, and an oxygen saturation of 100% on room air. On physical examination, she was alert, conversational, and in no acute distress. The patient had minimal tenderness in the lower abdomen, without rebound or guarding. Her lungs were clear with no increased work of breathing. The remainder of her exam was within normal limits. Treatment began with 1 liter bolus of crystalloid intravenous (IV) fluids, antiemetics, and pain control.

Overall, the initial laboratory studies were unremarkable, with a white blood cell (WBC) count of 8.42 10^3^ per microliter (10^3^/uL) (reference range 4-10 10^3^/uL), hemoglobin of 11.1 grams per deciliter (g/dL) (reference range 11.9-15.1 g/dL), hematocrit of 34.6% (35.5-44.9%), platelet count of 255 (140-440 10^3^/uL), creatinine 0.8 milligram per deciliter (mg/dL) (0.5-1.20 mg/dL), aspartate aminotransferase (AST) of 27 units per liter (10-55 U/L), alanine transaminase (ALT) of 20 (10-50 U/L), alkaline phosphatase of 51 (45-115 U/L), total bilirubin of 0.3 (0.1-1.2 mg/dL), and lipase of 23 (15-75 U/L). Her urine pregnancy test was negative. The urinalysis was significant for trace ketones, but no urinary tract infection. After the initial labs resulted, the patient reported persistent pain and had continued suprapubic tenderness on exam, so the decision was made to perform a computed tomography of the abdomen and pelvis (CT AP) with IV and oral contrast. 

Prior to going for imaging, the patient had a near syncopal episode while walking to bathroom. She was reevaluated and found to have an initial systolic blood pressure of 84 mmHg, but it improved to 95 mmHg within minutes without any intervention. At that time, a repeat glucose result of 67 mg/dL improved to 84 mg/dL after oral repletion. An additional 1 liter crystalloid fluid bolus was given with further improvement of her blood pressure.

**Figure 1:**
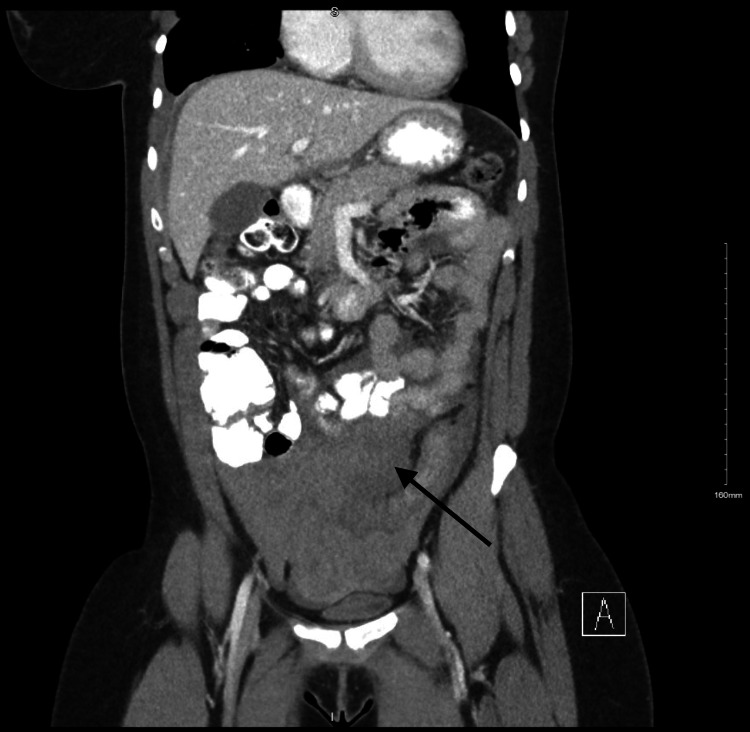
Computed tomography of the abdomen and pelvis (CT AP) with pelvic hematoma Coronal section of the CT AP performed on the day that the patient presented, showing a large volume of hemoperitoneum (arrow).

**Figure 2:**
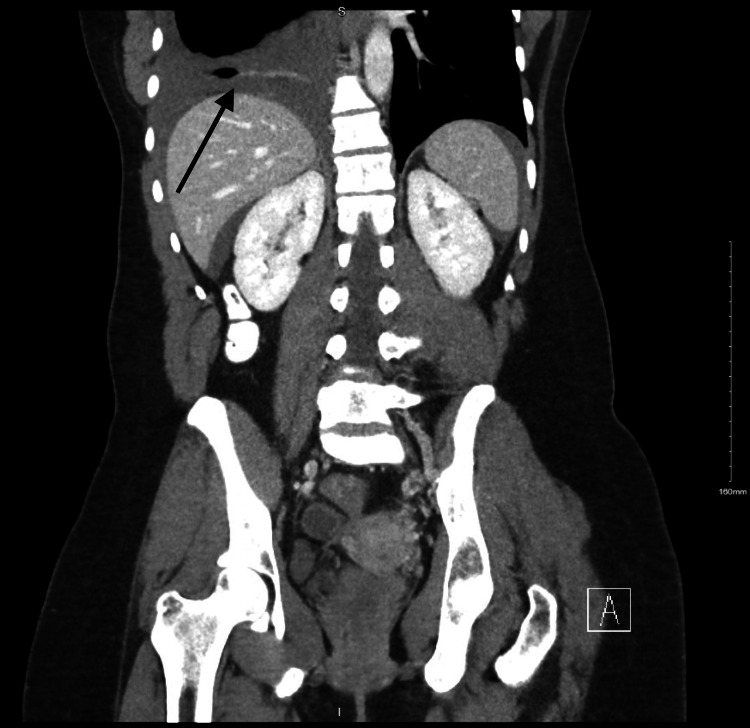
Computed tomography of the abdomen and pelvis (CT AP) with suspected hemothorax Coronal section of the CT AP performed on the patient's initial day of presentation with suspected right-sided hemothorax (arrow).

The CT AP was significant for a large pelvic hematoma measuring about 13 x 5.5 centimeters (cm) in axial dimension with active extravasation in the right adnexal region, concerning for a ruptured adnexal mass (Figure 1). Imaging also revealed a large right pleural effusion, slightly hyperdense, concerning for a hemorrhage tracking through a diaphragmatic defect (Figure 2).

She was evaluated emergently by gynecology, general surgery, and interventional radiology (IR). An additional large bore IV access was obtained, and she was consented for blood. Upon discussion with radiology, a CT angiogram of the chest, abdomen, and pelvis was performed to better evaluate for a source of active bleeding. This repeat imaging revealed no active arterial extravasation that IR could intervene upon. Repeat labs were significant for a WBC of 8.38 10^3^/uL, hemoglobin of 9.1 g/dL, hematocrit of 28.4%, and platelet count of 224.

The patient underwent an emergent diagnostic laparoscopy with gynecology and general surgery. She was found to have about 1 liter of dark red blood and clots throughout the abdomen, which was thought to be secondary to a ruptured ovarian cyst or ectopic endometrial tissue. In addition, the uterus and adnexa were densely adhered to the colon and pelvic wall. Endometrial lesions were found throughout the abdomen. No diaphragmatic injury was identified on direct inspection, and no specific area of bleeding was identified. The patient underwent lysis of adhesions and irrigation of her abdominal hematoma. Intraoperatively, the patient had a hemoglobin of 8 g/dL and required two units of packed red blood cell transfusions. No biopsies were performed during this procedure.

On post-operative day (POD) 1, the patient had a repeat chest X-ray (CXR) that showed a persistent right-sided pleural effusion. Thoracic surgery was consulted. Later that day, she underwent a right-sided chest tube placement, and about 1200 mL of hemorrhagic fluid was removed from the right chest upon placement. Over the next nine hours, the patient had 400 cc of serosanguinous output in the chest tube. The chest tube was removed on POD 3. Cytology on the pleural fluid demonstrated chronic inflammatory cells and reactive mesothelial cells, with no evidence for malignant cells or growth in aerobic or anaerobic cultures at three days. Additional fungal or atypical bacterial cultures were not performed on the chest tube output.

The patient was discharged on continuous oral contraceptive pills (OCPs) for menstrual suppression. The patient followed up with gynecology on POD 14 and was doing over all well, but she had some persistent cramps attributed to endometriosis.

## Discussion

While endometriosis is generally not a diagnosis that is made in the ED, the complications of endometriosis may be encountered by emergency clinicians. When endometriosis occurs outside of the pelvis, the most common location is in the thorax, typically presenting as a catamenial pneumothorax [3,6]. Thoracic endometriosis may also present as a hemothorax, hemoptysis, or even lung nodules [6]. Thoracic endometriosis is thought to be a secondary location, as patients typically also have pelvic endometriosis [6].

While the exact etiology of thoracic endometriosis is unknown, some theories have suggested that the pathogenesis could be related to coelomic metaplasia, spreading through the lymphatics or blood, or retrograde menstruation with endometrial tissue traveling into the thoracic cavity via defects in the diaphragm [3,4,6-10]. The diaphragmatic defects may be congenital or caused by previous endometrial implants on the abdominal aspect of the diaphragm [9,10]. Furthermore, the definitive cause of catamenial pneumothoraxes is unknown. It is thought to arise from intraabdominal endometriosis lesions spreading to the diaphragm, which creates perforations in the diaphragm secondary to hormone activation subsequently causing a pneumothorax [8]. Although there are other causes of catamenial pneumothoraxes, the main etiology of a catamenial hemothorax is thoracic endometriosis [4]. Thoracic endometriosis has been discovered in most, though not all, catamenial pneumothorax cases [3]. There is a debate in the literature regarding the association between pelvic endometriosis and catamenial pneumothoracies, although several articles find a strong correlation between catamenial pneumothoracies and pelvic endometriosis [3,6].

Catamenial pneumothorax is considered an uncommon type of spontaneous pneumothorax, often misdiagnosed as a spontaneous pneumothorax [7-8,11]. Prior studies cited the rates of spontaneous pneumothoraxes in females that meet the definition for catamenial pneumothoraxes to be around 3-6%, while more recent literature cites a higher rate, potentially up to 35% [7,11]. Most described cases of catamenial pneumothoraxes involve one lung, with about 85-95% on the right side [3,11]. About 75% of cases involve asymptomatic or mildly symptomatic pneumothoraxes [12].

Catamenial pneumothoraxes present with dyspnea, chest pain, cough, or shoulder pain within 24 hours prior to or up to 72 hours after a menstrual cycle [3,6-8,11]. Another defining characteristic is the lack of symptoms outside of menstruation [12]. Asking history questions related to the timing of woman’s menstrual cycle may help reveal this diagnosis [11]. Catamenial pneumothorax must be considered for any menstruating female presenting with reoccurring pneumothoraxes [12].

Endometriosis does not have a definitive cure, but it can be managed medically and surgically [4]. Treatment for thoracic endometriosis centers around the extent of the disease. When thoracic endometriosis is managed with medical therapy alone, about 60% of patients may have recurrence within one year [4]. Due to the suggested correlation of catamenial pneumothoraxes with endometriosis, a multidisciplinary approach with gynecologists, thoracic surgeons, and pulmonologists will likely aid in the development of an effective treatment strategy [6]. For acute and severe cases, thoracic endometriosis will generally go to the operating room for a thoracoscopy [6].

When initially managing catamenial pneumothoraxes, the treatment is similar to typical pneumothoraxes [11,12]. Asymptomatic or mildly symptomatic catamenial pneumothoraxes can be managed conservatively, while more symptomatic presentations will likely need drainage [6,12]. The high recurrence rate of catamenial pneumothoraxes is the challenge in devising an effective long-term management strategy [8]. Surgical intervention may be required to avoid recurrence [3,11]. One source cites that the most ideal time for surgery is during menstruation to be able to see the pathology [8]. A combination of surgical treatment and hormonotherapy is likely to yield an improved outcome [3,6,7].

## Conclusions

Although catamenial pneumothorax is an uncommon diagnosis, it is important for emergency physicians to consider it as a cause of spontaneous pneumothoraxes in menstruating females. As in this case, there is potential for either hemorrhagic or obstructive shock, which will require emergency resuscitation. Although initial presentation may be complicated by the identification of critical illness, endometriosis is a diagnosis that may bring a patient to the ED.
